# Determination of Shear Strength Parameters of Concrete Materials Based on the Rectangular Section Splitting Method

**DOI:** 10.3390/ma17246141

**Published:** 2024-12-16

**Authors:** Jinchao Yue, Da Wang, Yan Jiang, Shoukun Shi, Yibin Huang

**Affiliations:** 1 Yellow River Laboratory, Zhengzhou University, Zhengzhou 450001, China; yuejc@zzu.edu.cn (J.Y.); wd20001022@163.com (D.W.); benbenjiangyan@163.com (Y.J.); 13087025558@163.com (S.S.); 2 Geotechnical Engineering Research Institute, Guangdong Research Institute of Water Resources and Hydropower, Guangzhou 510610, China

**Keywords:** rectangular section splitting method, concrete, Mohr–Coulomb formula, cohesion, friction angle, finite element simulation

## Abstract

This paper introduces an alternative method for determining the shear strength parameters of concrete materials, specifically the rectangular section splitting method, to ascertain the shear strength parameters of concrete materials. Based on the Mohr–Coulomb failure criterion, formulas for calculating the cohesion (c) and the angle of internal friction (φ) of concrete materials are derived. Numerical simulation is employed to fit and solve for the coefficients involved in the formulas. Subsequently, the concrete rectangular section splitting method and direct shear tests are utilized to verify the derived formulas. The results indicate that there is a certain feasibility to the shear strength parameters obtained for concrete materials through the rectangular section splitting method. The cohesion (c) differs by approximately 3.65%, and the angle of internal friction (φ) differs by about 6.94% when compared to the shear strength parameters obtained through direct shear tests. This suggests that the rectangular section splitting method provides a viable approach for determining the shear strength parameters of concrete materials.

## 1. Introduction

The splitting test is primarily utilized for measuring the tensile strength of brittle materials. Compared to traditional direct tension tests, it offers the advantages of simplicity in operation and minimal deviation coefficients. The types of specimens for splitting tests can be selected as circular or rectangular sections; the circular section is known as the Brazilian splitting test. To ensure reliable results in the Brazilian splitting test, it is crucial to maintain uniform tensile stress on the splitting plane and avoid eccentric loading [1,2,3]. This test demands high standards in the collection and fabrication of test blocks, as well as in the application of loads. The rectangular section splitting test is more convenient in terms of specimen preparation and loading compared to the circular section splitting test. Additionally, the shear-strength parameters such as cohesion (c) and the angle of internal friction (φ) of concrete can be assessed based on geotechnical strength criteria to determine the safety of stress–strain conditions in geotechnical engineering. These parameters are typically required with specific values in numerical modeling. The existing determination of concrete shear-strength parameters is generally conducted through triaxial tests, where the Mohr–Coulomb law is applied to fit and calculate the data [4,5]. This process is more complex than the splitting test and requires specific rock-direct shear equipment. If it were possible to obtain the cohesion (c) and the angle of internal friction (φ) of concrete materials through common testing equipment like the rectangular section splitting test, it would significantly simplify the process.

Currently, to determine the shear-strength parameters of geotechnical materials, numerous scholars have proposed various methods for measuring the shear-strength parameters of such materials. Labuz, J.F. [6], based on the Mohr–Coulomb failure criterion, derived formulas for calculating the cohesion (c) and the angle of internal friction (φ) by solving the stress envelope of triaxial tests and verified these through experiments; Seung-Jun Kwon [7] proposed an integrated model for the shear strength of concrete interfaces from the perspective of the plastic limit theory of concrete. This model takes into account the influence of additional axial stress and lateral reinforcement on the shear friction at the interface shear crack, and its reliability was verified by comparing it with previous empirical formulas and 103 different push-off test samples from various literature sources; Lelovic, S. [8] introduced a novel method for determining cohesion at preset shear deformation angles, which involves first measuring the indirect tensile strength of the material, then calculating the Mohr circle values and determining the cohesion as the shear stress value on the Mohr circle where the normal stress is zero. In the numerical computation section, the shear friction angle from the Mohr–Coulomb (MC) model was used, and the ultimate load theory was applied to the Brazilian test model, matching experimental results with numerical calculations to verify the applicability of this method in numerical analysis; Komurlu, E. [9] used the double shear jaws (DSJ) test to determine the cohesion values of different types of rock materials through experimental and numerical analysis and investigated the effects of different test conditions such as jaw-plate size, three-block gap, and jaw-plate contact conditions on the cohesion test results and validity; Lelovic, S [10] proposed a straightforward method for determining two material parameters of concrete, cohesion (c) and internal friction angle (φ), based on indirect tension tests and a theoretical framework. Cohesion (c) and internal friction angle (φ) were derived from experimental results of concrete deformation within the realms of both elastic and plastic theories. Numerical analysis was conducted using the finite element method, and the boundary value problems were solved using a step-by-step integration approach to validate the experimental outcomes. Gong, F. [11] conducted preset angle shear tests, direct shear tests, and triaxial compression tests on two representative rocks, marble and red sandstone. The cohesion (c) and internal friction angle (φ) under different test conditions were determined, and the stress–strain relationships, failure modes, and shear-strength parameters of the marble and red sandstone samples were analyzed and compared. Based on the test results, a rational method for estimating shear-strength parameters was proposed. Ebrahim A.S, A [12] introduced a micro-triple-axis test (MTT) device and used it as an index test to apply triaxial loads to small cubic rock samples to estimate their shear-strength parameters. The repeatability of the results was determined by conducting 27 MTTs on 6 × 6 × 6 mm polymethyl methacrylate (PMMA) cubic samples. The results showed that the values measured by the device were repeatable, with a similarity exceeding 97%. The internal friction angles (φ) obtained from conventional triaxial tests (CTTs) on cylindrical samples and a MTT on 6 × 6 × 6 mm samples for mortar samples were similar, thereby proving the feasibility of using small-scale triaxial tests to estimate the shear-strength parameters of intact limestone.

In addition to determining material shear-strength parameters through improved testing techniques, some scholars have also used machine learning to establish predictive models for determination. Shahani, N.M [13] developed four advanced machine learning-based intelligent predictive models for estimating the cohesion (c) and internal friction angle (φ) of rocks, using P-wave velocity, density, uniaxial compressive strength, and tensile strength as input parameters. An iterative five-fold cross-validation method was employed, with the coefficient of determination, mean absolute error, mean squared error, and root mean squared error serving as performance metrics to evaluate the optimal predictive model. The results indicated that uniaxial compressive strength and tensile strength are the parameters with the greatest influence on predicting cohesion (c) and internal friction angle (φ). The findings of this study contribute to the assessment of long-term stability and deformation of rock masses in surface and underground excavations. Fathipour-Azar, H. [14] employed computational data analysis methods to propose a novel technical approach for measuring the strength parameters of geomaterials. The study primarily utilized Random Forest, Alternating Model Trees, and Support Vector Machine (SVM) technologies to predict the Mohr–Coulomb shear-strength parameters of geomaterials. The models were trained and tested using a published database of geomaterials, including uniaxial compressive strength, uniaxial tensile strength, and various stress conditions. The average error of the established cohesion strength prediction model was less than 1.1 during both training and testing phases, and the average root mean square error was less than 1.5 MPa, demonstrating the effectiveness of this method in predicting the shear-strength parameters of sandstone. This research outcome aids in the long-term stability and deformation assessment of rock masses in surface and underground excavations. Danial, J. A. [15] proposed an indirect method for measuring the shear-strength parameters of shale through stratigraphic index testing. Triaxial compression tests were conducted to obtain the shear-strength parameters of 230 samples, along with relevant rock parameters. A Particle Swarm Optimization Artificial Neural Network (PSO-ANN) integrated model was established, using the results of rock index tests as inputs and shear-strength parameters as the model’s output. The determination coefficients of 0.966 and 0.944 obtained for the training and testing datasets indicate that the model can be used to predict the shear-strength parameters of shale with high precision.

However, these research contents all pose certain difficulties in terms of experimental equipment and implementation processes, and a series of challenging issues arise during the specific operation. Based on this, the present study aims to combine common splitting tests with numerical simulations to develop a method for determining the shear-strength parameters of geotechnical materials such as concrete and to provide relevant formulas. This approach can further ascertain their shear strength and simplify the process of determining the shear-strength parameters of concrete materials, while also obtaining their tensile strength. For geotechnical materials, which exhibit good compressive properties but poor tensile performance, the main causes of failure are the materials’ tensile strengths and linear strains reaching their ultimate limits. The failure theories are primarily the first strength theory, which is the maximum tensile stress theory, and the second strength theory, which is the maximum tensile strain theory [16,17]. The rectangular section splitting method can obtain the tensile stress considered in the strength theory and indirectly measure its shear-strength parameters. This is of reference value for evaluating material characteristics and their applicable scope, providing a basis for the verification of structural strength, stiffness, and stability.

The study on determining the shear-strength parameters of concrete using the rectangular section splitting method introduces a novel testing approach for assessing the shear-strength parameters of concrete and other analogous materials. This research is of significant theoretical importance and practical value for the further refinement of mechanical performance testing for concrete materials. Consequently, it serves as a reference for researchers both domestically and internationally who are involved in the investigation of splitting tensile strength.

## 2. The Basic Principle of the Rectangular Section Splitting Method

The shear strength of certain geological materials, such as soil and rock, is typically determined by the Mohr–Coulomb failure theory, which encompasses two parameters: the material’s cohesion (c) and the angle of internal friction (φ). These parameters are generally established through indoor triaxial tests or direct shear tests on rock. The Mohr–Coulomb theory is extensively applied to determine the shear strength of soils and is also suitable for cement-stabilized soils and granular base courses. However, triaxial tests require very expensive equipment and a significant amount of time for sample preparation and testing, making them unsuitable for routine experiments or field tests. A novel approach is proposed to determine the shear-strength parameters of concrete materials using the rectangular section splitting method, which combines the rectangular section splitting test with the unconfined compressive strength test. By employing the Mohr–Coulomb theory, this method aims to determine the cohesion and the angle of internal friction of concrete materials.

**Figure 1:**
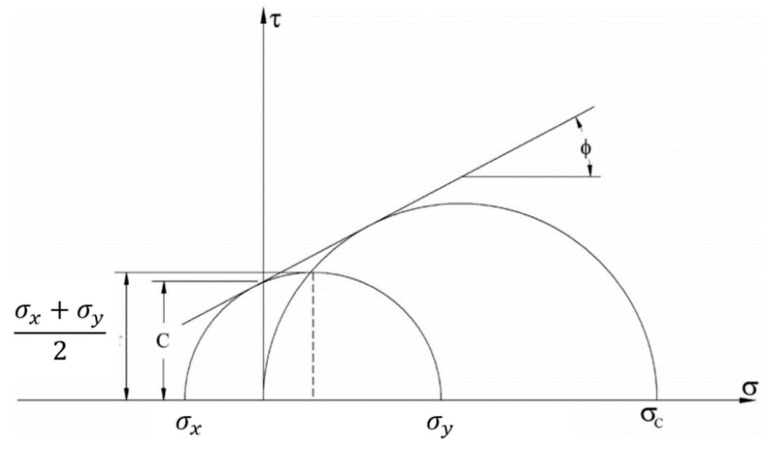
Mohr’s circle for splitting test and unconfined compressive strength test.

The Mohr–Coulomb theory is represented by Mohr’s circles, where a series of circles depicting the stress state at the initial stage of failure under increasing confining pressure are plotted. The common tangent to these Mohr’s circles is then drawn, and the cohesion (c) and the angle of internal friction (φ) of the material are determined from this tangent. By combining the splitting test and the unconfined compressive strength test, Mohr’s circles are constructed as shown in Figure 1.

From Figure 1, it can be observed that:

(1) $$\frac{\sigma_{y}-\sigma_{x}}{2}=(c\cot\varphi +\frac{\sigma_{x}+\sigma_{y}}{2})\sin\varphi$$

(2) $$\frac{\sigma_{c}}{2}=(\frac{\sigma_{c}}{2}+c\cot\varphi )\sin\varphi$$

(3) $$(1),(2)\to \cot\varphi =\frac{\sigma_{y}-\sigma_{x}}{2\sin\varphi }-\frac{\sigma_{x}+\sigma_{y}}{2}=\frac{\sigma_{c}}{2\sin\varphi }-\frac{\sigma_{c}}{2}$$

where *σ_c_* is the unconfined compressive strength of concrete, *σ_x_* is the tensile stress at the center of the concrete specimen, σ_y_ is the compressive stress at the center of the concrete specimen, c is the cohesion stress of the concrete material, and φ is the internal friction angle of the concrete.

Huang, Y.G. [18] proposed that during the splitting process of rock-like materials, there is a corresponding relationship between the tensile stress and compressive stress at the center of the specimen, as well as between the splitting strength of the specimen and the horizontal tensile stress at the center. Assuming the relationship between the splitting strength (*σ_IDT_*) of the specimen and the horizontal tensile stress (*σ_x_*) at the center, with a modification factor of k*, the equation is as follows:

(4) $$\sigma_{x}=k^{*}\sigma_{IDT}$$

The relationship between the horizontal tensile stress (*σ_x_*) and the compressive stress (*σ_y_*) at the center of the specimen is:

(5) $$\sigma_{y}=k_{s}\sigma_{x}$$

(6) $$(4),\;(5)\to \sigma_{y}=k_{s}\sigma_{x}=k_{s}k^{*}\sigma_{IDT}$$

where *σ_IDT_* is the splitting strength of concrete,$\;k^{*}\;$is the influence coefficient between the splitting strength (*σ_IDT_*) of the specimen and the horizontal tensile stress (*σ_x_*) at the center, and $k_{s}\;$is the influence coefficient between the horizontal tensile stress (*σ_x_*) and the compressive stress (*σ_y_*) at the center of the specimen.

Equation (6) is substituted into Equation (3) to solve, and then:

(7) $$\sin\varphi =\frac{k^{*}(k_{s}-1)\sigma_{IDT}-\sigma_{c}}{k^{*}(1+k_{s})\sigma_{IDT}-\sigma_{c}}$$

(8) $$c=\frac{\sigma_{c}}{2\cot\varphi }(\frac{1}{\sin\varphi }-1)=\frac{\sigma_{c}}{\cot\varphi }\cdot \frac{k^{*}\sigma_{IDT}}{k^{*}(1+k_{s})\sigma_{IDT}-\sigma_{c}}$$

Utilizing the aforementioned theory to determine the cohesion (c) and the angle of internal friction (φ) of the material, it is only necessary to measure the unconfined compressive strength and the splitting strength of the specimen to obtain the shear-strength parameters. This method is simple and efficient, and it does not require complex triaxial testing equipment or other specialized devices.

## 3. Stress Relationship in Splitting Test Specimen

From the theoretical derivations mentioned above, it can be deduced that to obtain the shear-strength parameters of concrete materials through the rectangular section splitting method, one must first establish the relationship between the tensile stress and compressive stress at the center of the specimen and the specimen’s splitting strength. That is, it is necessary to determine the correction factor ($k^{*}$) for the relationship between the splitting strength (*σ_IDT_*) and the horizontal tensile stress at the center (*σ_x_*), as well as the influence factor ($k_{s}$) for the relationship between the horizontal tensile stress at the center (*σ_x_*) and the compressive stress (*σ_y_*). The determination of $k_{s}$ and $k^{*}$ will now be discussed. SłowiK, M. [19] conducted a study on the influence of cylindrical specimen length on the tensile strength of concrete determined by the Brazilian splitting test. The experiment involved two different sizes of cylindrical specimens with a diameter of 150 mm and lengths of 150 mm and 300 mm, respectively, to test the tensile strength of concrete. Large-scale statistical analysis was performed using the Statistica(version 13, StatSoft Polska Sp. z o.o, Krakow, Poland) software. The results indicated that the shape and size of the specimens have a certain impact on the splitting tensile strength. Yong Yu [20] utilized discrete element simulations to investigate the effects of different water–cement ratios on the compressive strength, elastic modulus, peak strain, and failure modes of Recycled Aggregate Concrete (RAC). The conclusions drawn were that RAC is influenced by size effects, albeit not as significantly as Normal Aggregate Concrete (NAC) with the same water–cement ratio, but more so than mortar. Deng Huafeng [21] carried out splitting tensile strength tests on over 100 sandstone disc specimens with varying thickness-to-diameter ratios. The test results demonstrated that as the thickness-to-diameter ratio decreased, the tensile strength gradually increased. When the thickness-to-diameter ratio was approximately less than 0.3, the tensile strength increased and approached a relatively stable value. Three-dimensional finite element models with different thickness-to-diameter ratios were also established to study the distribution patterns of equivalent stress along the central axis of the discs in detail. The results showed that the maximum tensile stress along the central axis of the specimens occurred at the center of the specimen’s end faces, and the larger the thickness-to-diameter ratio of the specimens, the greater the tensile stress at the midpoint of the disc’s end face. Ince, R. [22] conducted Brazilian splitting tests on cubic and diagonal cubic specimens with similar shapes but different sizes, considering various load distribution methods. The test results were analyzed using a modified size effect law, a predictive formula was proposed, and historical test data were compared to ascertain that the size effect of concrete and the loading method have a significant influence on the test outcomes.

Equation (5) assumes a coefficient relationship between compressive stress and tensile stress, denoted as $k_{s}$. Based on existing research outcomes [23,24,25,26], it can be derived that for disk-shaped specimens, the compressive stress at the center is three times the tensile stress. For rectangular cross-section specimens, this ratio varies and is primarily influenced by the specimen’s loading width (i.e., ratio of the bearing strip), the aspect ratio of height to length, and the thickness. To obtain an expression for the influence coefficient $k_{s}$, the effects of loading width, aspect ratio, and thickness on the correction coefficient were considered. Finite element software(Abaqus/CAE 2022) was utilized for simulation, and fitting curves were further plotted to provide the corresponding formulas for each factor’s impact on $k_{s}$.

### 3.1. Numerical Modeling

**Figure 2:**
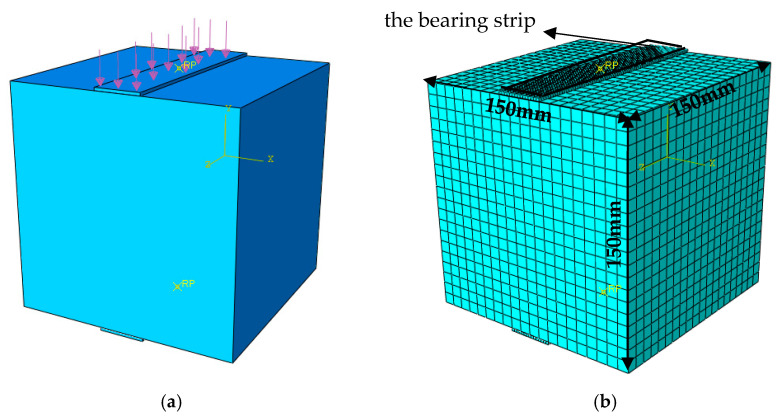
Finite element calculation model: (**a**) is the loading case, (**b**) is the grid division case.

The numerical simulation process is conducted using the Abaqus/CAE 2022 finite element software for splitting tests. The material selected for analysis is C35 concrete. The applied load is in the form of a distributed load, with a design value of 20 kN, directed vertically downward. The specific load application is as shown in Figure 2a. The finite element model uses a cubic specimen with dimensions of 150 mm × 150 mm × 150 mm, with a pad width of 30 mm; thus, the pad ratio is set to 0.2. The pad is modeled as a rigid body, and the interaction between the pad and concrete is achieved through contact. The tangential behavior of the contact is set to rough contact, while the normal behavior is set to hard contact. The Poisson’s ratio for concrete is taken as 0.2, and the elastic modulus is set to 3.0 × 10^3^ MPa. The Mohr–Coulomb elastoplastic model is adopted for the material. The material density is 2200 kg/m^3^. The cohesion of the material is set to 4.81 MPa, and the internal friction angle is set to 55°. The element type used is C3D20, and the finite element model is as shown in Figure 2b.

### 3.2. Effect of Bearing Strip Ratio on k_s_

Considering the influence of the distributed load width on the splitting tensile strength ($k_{s}$), i.e., the effect of the pad ratio on $k_{s}$, common splitting test loading conditions are simulated numerically. In the numerical simulation process, the pad ratio is set to 0.1, 0.15, 0.2, 0.25, 0.3, 0.4, and 0.5, corresponding to pad widths of 15 mm, 22.5 mm, 30 mm, 37.5 mm, 45 mm, 60 mm, and 75 mm, respectively. The numerical simulation results extract the numerical relationship between the compressive stress and tensile stress ratio at the center of the specimen, as shown in Table 1.

**Figure 3:**
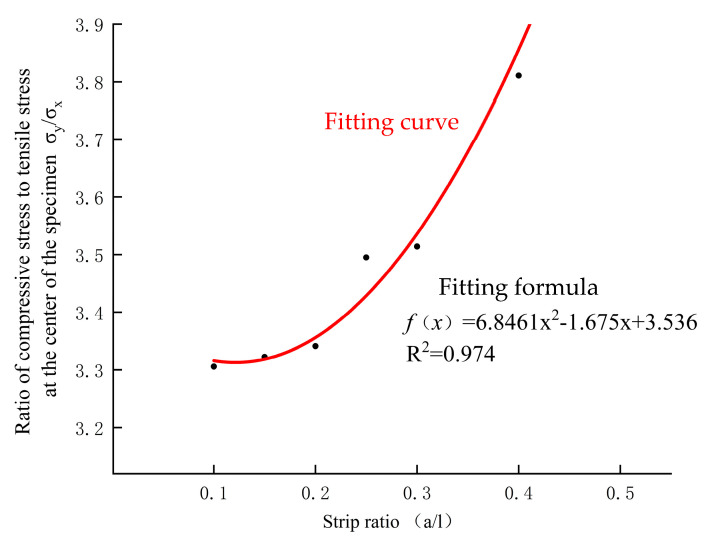
Relation between stress ratio and strip ratio.

The data in Table 1 were drawn and fit, and the fitting results are shown in Figure 3.

From the fitting of Figure 3, it can be observed that the stress ratio at the center of the specimen has a quadratic relationship with the strip ratio *a/l*. The stress correction coefficient $k_{s-s}$ due to the loading width is hypothesized to have the following expression:

(9) $$k_{s-s}=\frac{1}{3}(6.8461{\left(\frac{a}{l}\right)}^{2}-1.675\left(\frac{a}{l}\right)+3.536)$$

### 3.3. Effect of Specimen Height-to-Length Ratio on k_s_

Considering the influence of the aspect ratio on the stress distribution within the specimen, based on common splitting test loading conditions, the aspect ratio is set to 0.25, 0.5, 0.75, 1.0, 1.5, and 2.0 in the numerical simulation process. This corresponds to specimen heights of 37.5 mm, 75 mm, 112.5 mm, 150 mm, 225 mm, and 300 mm, respectively. The relationship between the compressive stress and tensile stress ratio at the center of the specimen is extracted and presented as shown in Table 2.

**Figure 4:**
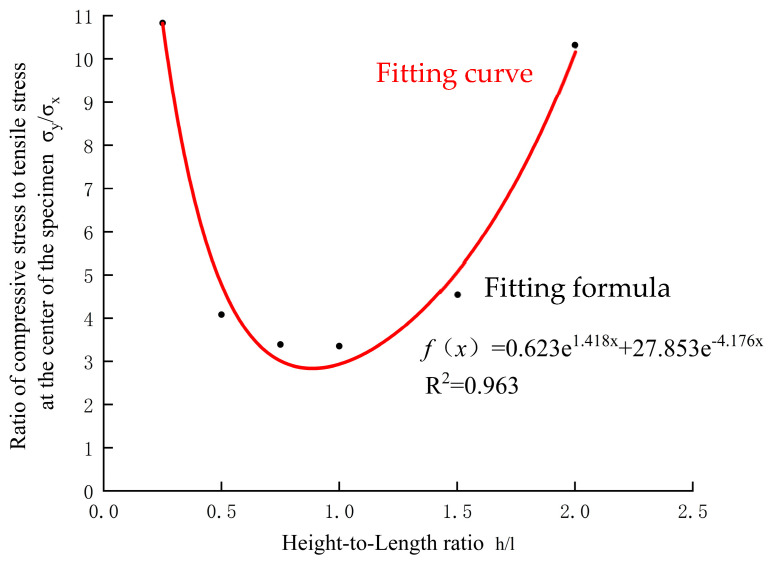
Relation between stress ratio and height-to-length ratio h/l.

The data in Table 2 were drawn and fit, and the fitting results are shown in Figure 4.

From Figure 4, it is evident that the stress ratio at the center of the specimen has a complex relationship with the height-to-length ratio h/l, generally following an exponential curve. When the height-to-length ratio approaches 1.0, the stress ratio at the center of the specimen is at its minimum. The stress correction coefficient $k_{s-h}$, due to different height-to-length ratios of the specimens, is hypothesized to have the following expression:

(10) $$k_{s-h}=\frac{1}{3}(0.623e^{1.418h/l}+27.853e^{-4.176h/l})$$

### 3.4. Effect of Specimen Thickness on k_s_

Considering the impact of thickness on the stress distribution within the specimen, based on common splitting test loading conditions, the thickness-to-length ratio is set to 0.25, 0.5, 0.75, 1.0, 1.5, 2.0, 2.5, and 3 in the numerical simulation process. This corresponds to specimen thicknesses of 37.5 mm, 75 mm, 112.5 mm, 150 mm, 225 mm, 300 mm, 375 mm, and 450 mm, respectively. The relationship between the compressive stress and tensile stress ratio at the center of the specimen is extracted and presented as shown in Table 3.

**Figure 5:**
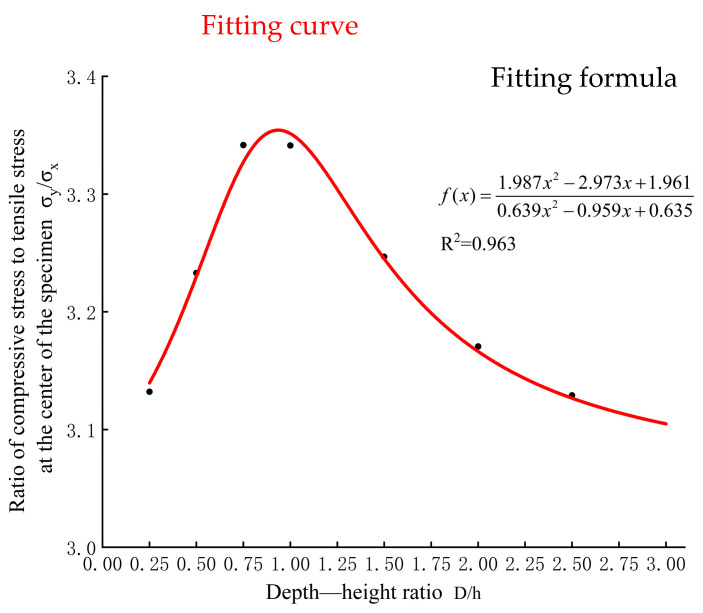
Relation between stress ratio and height-to-length ratio D/h.

Table 3 is drawn, and the data in it are fitted, and the fitting results are shown in Figure 5.

From Figure 5, it can be observed that the relationship between the stress ratio at the center of the specimen and the thickness is quite complex. The stress ratio is maximum when the thickness-to-height ratio is 1. The rate of decrease in the stress ratio varies with different thicknesses. The stress correction coefficient $k_{s-d}$ due to the specimen thickness is hypothesized to have the following expression:

(11) $$k_{s-d}=\frac{1}{3}\frac{1.987{\left(\frac{D}{h}\right)}^{2}-2.973(\frac{D}{h})+1.961}{0.639{\left(\frac{D}{h}\right)}^{2}-0.959(\frac{D}{h})+0.635}$$

Taking into account all the influencing factors, the correction coefficient $k_{s}\;$for the relationship between the compressive stress and tensile stress at the center of the splitting test specimen can be expressed as:

(12) $$k_{s}=3k_{s-s}k_{s-h}k_{s-d}$$

### 3.5. Determination of Correction Coefficient k*

When conducting splitting tests on specimens, the tensile stress generated at the center of the specimen under the action of external loads exhibits a certain relationship in materials of the same nature and under the action of external loads. The value of this tensile stress (*σ_x_*) can be expressed as:

(13) $$\sigma_{x}=k^{*}\sigma_{IDT}=k^{*}\frac{2P}{\pi A}$$

where *σ_IDT_* is the splitting tensile strength of old and new concrete (MPa), P is tensile failure load (N), and A is the area of the specimen’s splitting surface (mm^2^).

The relationship between the splitting strength (*σ_IDT_*) of the specimen and the horizontal tensile stress at the center, considering various influencing factors, is assumed to have a correction factor $k^{*}$. Numerical simulations are conducted by altering the magnitude of different applied loads, and the calculated results are presented in Table 4.

From Table 4, it can be observed that under different loading conditions, the relationship between the tensile stress at the center of the specimen and the splitting strength remains approximately constant. $k^{*}\;$can be approximated as 0.885.

## 4. Determination of Material Parameters and Verification

Through the derivation of the theoretical formulas for the rectangular section splitting method, the relevant coefficients were provided with corresponding calculation formulas by numerical simulation, leading to the determination of the shear-strength parameters of concrete materials using the rectangular section splitting method. Currently, based on the available experimental equipment, the rectangular section splitting method and shear tests are conducted on concrete to determine the shear-strength parameters of the material and to verify the rationality of this method.

### 4.1. Experimental Design

The concrete is prepared using grade 52.5 ordinary Portland cement, medium-coarse river sand (fineness modulus of 2.73), and crushed stone (5–20 mm). The designed grade of the concrete is C35, with the concrete dimensions being 150 mm × 150 mm × 150 mm. The mix design for the concrete material is shown in Table 5.

### 4.2. Rectangular Section Splitting Method

**Figure 6:**
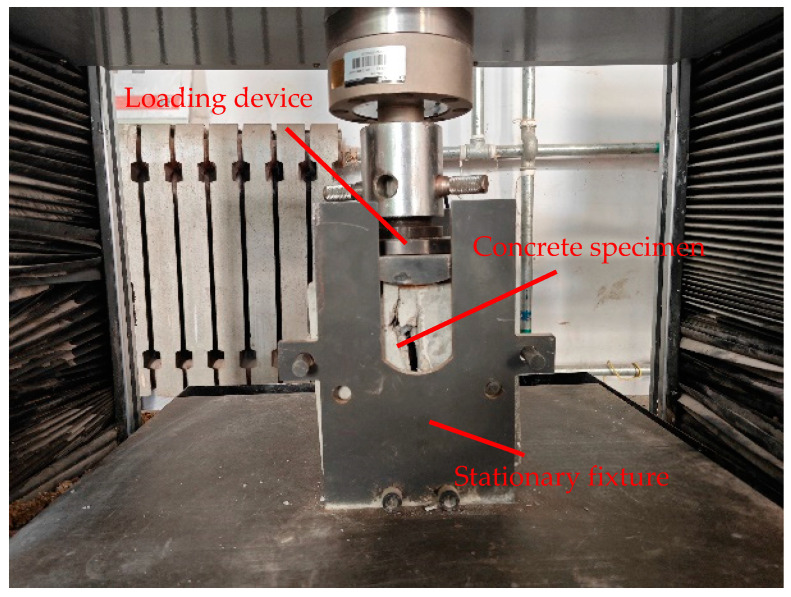
Schematic diagram of splitting test.

**Figure 7:**
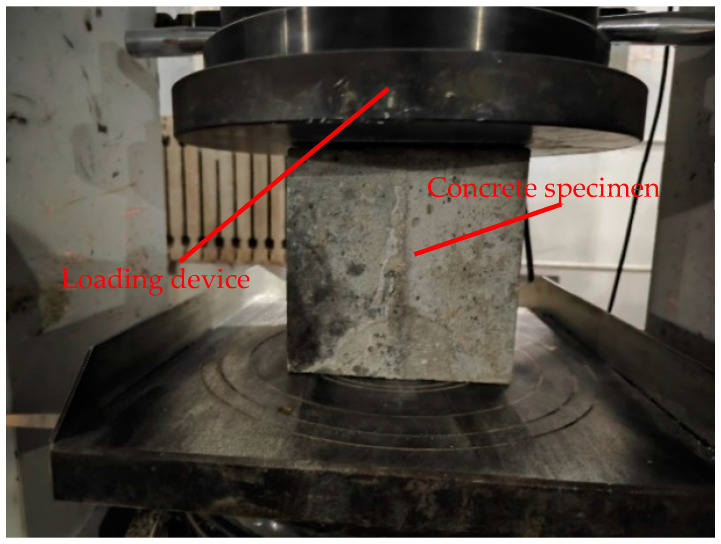
Schematic diagram of unconfined compression test.

The concrete splitting test is conducted in accordance with the splitting test specifications [27]. The support pad, made of hard three-ply board, is 30 mm wide and 2 mm thick. A universal testing machine is used to perform the splitting test, and the loading method is as shown in Figure 6 and Figure 7.

Using load control, the loading rate is set to 0.02~0.05 MPa per second, applying the load continuously and uniformly until the specimen fails. The failure load of the specimen is recorded, and the splitting strength (*σ_IDT_*) is calculated according to Equation (14).

(14) $$\sigma_{IDT}=\frac{2P}{\pi A}=0.637\frac{P}{A}$$

where *σ_IDT_* is the splitting tensile strength of old and new concrete (MPa), P is tensile failure load (N), and A is the area of the specimen’s splitting surface (mm^2^).

The unconfined compressive strength test of concrete is conducted in accordance with the compressive test specifications [27]. A rock compressive strength testing machine is used to determine the strength, and the loading situation is as shown in Figure 7.

Using load control, the loading rate is set to 0.5~0.8 MPa per second, applying the load continuously and uniformly until the specimen fails. The compressive strength (*σ_c_*) of the specimen is recorded.

The final test results are shown in Table 6:

It can be seen from the test that the ratio of the pad strip is 0.2, the ratio of height to length of the specimen is 1, and the ratio of thickness to height is 1, which can be obtained by substituting Equations (9)–(11) with $k_{s}=3k_{s-s}k_{s-h}k_{s-d}=3\times 1.158\times 1.0\times 0.976=3.391$, and by substituting $k^{*}=0.885$ into the Equations (7) and (8) can be obtained:

$$\sin\varphi =\frac{\sigma_{c}-k^{*}(k_{s}-1)\sigma_{IDT}}{\sigma_{c}-k^{*}(1+k_{s})\sigma_{IDT}}=0.78,c=\frac{\sigma_{c}}{\cot\varphi }\cdot \frac{k^{*}\sigma_{IDT}}{k^{*}(1+k_{s})\sigma_{IDT}-\sigma_{c}}=4.636$$

Finally, the cohesive force (c) of C35 concrete is 4.636 MPa, and the internal friction angle (*φ*) is 51.583°.

### 4.3. Verification of Shear-Strength Parameters of Concrete

**Figure 8:**
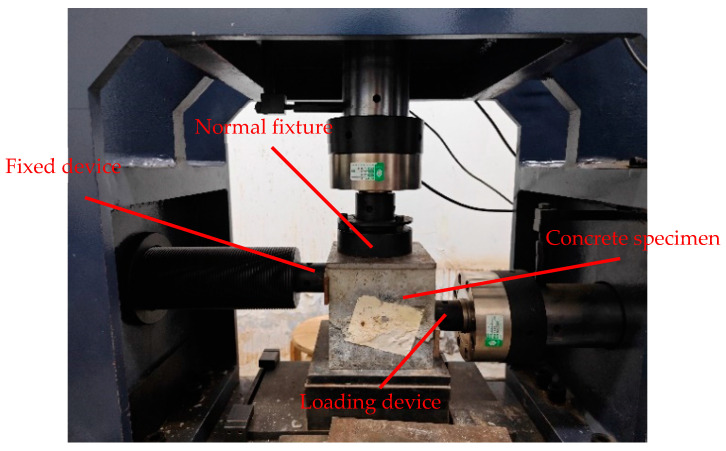
Schematic diagram of concrete direct shear test.

In order to verify the rationality of the above method, direct shear test of concrete is carried out in accordance with the specifications of the shear test in [28]. The test device is a rock-direct shear instrument, and the loading mode of the direct shear test is shown in Figure 8.

In the direct shear test, the normal pressures are set to 1 MPa, 2 Mpa, 3 Mpa, and 4 Mpa, and the corresponding shear failure tangential pressures are recorded. The shear test results are then fitted to data, and the cohesion © and the angle of internal friction (*φ*) of the specimen are obtained using the Mohr–Coulomb formula τ=σtanφ+c. The direct shear test and fitting results are shown in Table 7.

**Figure 9:**
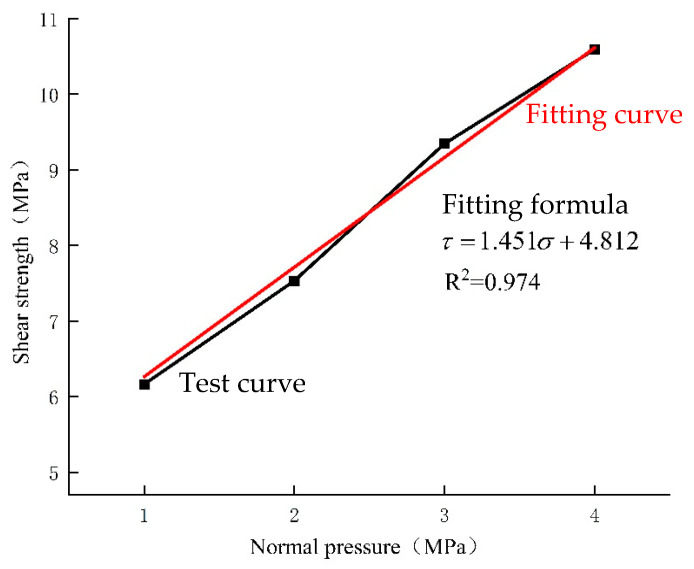
Fitting image of concrete direct shear data.

And the fitting is plotted in Figure 9.

From the above, it can be obtained that the cohesion (c) of C35 concrete determined by the rectangular section splitting method is 4.636 MPa, and the angle of internal friction (*φ*) is 51.583°. In contrast, the direct shear test yields a cohesion (c) of 4.812 MPa and an angle of internal friction (*φ*) of 55.43° for C35 concrete. The difference in cohesion (c)between the two methods is approximately 3.65%, and the difference in the angle of internal friction (*φ*) is approximately 6.94%. Selim P. [29] determined the cohesion (c) of C35 concrete to be 4.72 MPa and the angle of internal friction (*φ*) to be 55.31°. Comparing these results with those obtained by the rectangular section splitting test method, the difference in cohesion (c) is approximately 1.78%, and the difference in the angle of internal friction (*φ*) is approximately 6.74%. This confirms the rationality of using the rectangular section splitting method to determine the shear-strength parameters of concrete materials.

## 5. Conclusions

The shear-strength parameters of concrete materials can be used to assess whether the stress–strain in geotechnical engineering is safe according to geotechnical strength criteria, thereby evaluating the material’s shear resistance. Research on the rectangular section splitting method to determine the shear-strength parameters can simplify the process of obtaining shear-strength parameters through conventional direct shear tests on soil and rock. This paper derives the basic formulas for determining the shear-strength parameters of concrete materials using the rectangular section splitting method through theoretical formula derivation, combines numerical calculations to fit the relevant coefficients involved in the formulas, and verifies the method through concrete direct shear tests. The following conclusions are drawn:

(1) Through the derivation of the Mohr’s circle formulas, the theoretical basis for determining the shear-strength parameters of materials using the rectangular section splitting test is obtained, and specific formulas are provided;
(2) There is a certain correlation between the compressive stress and tensile stress at the center of the rectangular concrete section and the splitting strength of the specimen. The ratio of compressive stress to tensile stress at the center of the specimen, as well as the influence of the stiffener ratio, height-to-length ratio, and thickness on this ratio, can be determined through finite element simulation based on the splitting test;
(3) The shear-strength parameters of concrete materials were measured by the rectangular section splitting method and compared with the results measured by direct shear test. It was found that the difference in the cohesive force (c)and the difference in the internal friction angle (*φ*) between the two test results was about 7.64%, and the difference in the internal friction angle φ was about 5.85%. The comparison of test results showed that the theory was reasonable.

## Figures and Tables

**Table 1:** Effect of bearing strip ratio on stress ratio at the center of specimen.

Bearing Strip Ratio a/l	Stress in the x-Direction at the Center *σ_x_* (MPa)	Stress in the x-Direction at the Center *σ_y_* (MPa)	*σ_y_*/*σ_x_*
0.1	0.49223	−1.62717	3.30574
0.15	0.48256	−1.59850	3.32256
0.2	0.46040	−1.54293	3.34128
0.25	0.41583	−1.45342	3.49522
0.3	0.41074	−1.44341	3.51419
0.4	0.35104	−1.33779	3.81092

**Table 2:** Effect of the height-to-length ratio h/l on the stress ratio at the center of the specimen.

Height-to-Length Ratio h/l	Stress in the x-Direction at the Center *σ_x_* (MPa)	Stress in the x-Direction at the Center *σ_y_* (MPa)	*σ_y_*/*σ_x_*
2.0	0.08917	−0.92058	10.32433
1.5	0.24271	−1.08048	4.55173
1.0	0.46040	−1.54293	3.35128
0.75	0.53123	−1.96041	3.39032
0.5	0.61868	−2.58892	4.0846
0.25	0.34714	−3.79492	10.83187

**Table 3:** Effect of the thickness-to-height ratio D/h on the stress ratio at the center of the specimen.

Thickness-to-Height Ratio D/h	Stress in the x-Direction at the Center *σ_x_* (MPa)	Stress in the x-Direction at the Center *σ_y_* (MPa)	*σ_y_*/*σ_x_*
3	0.11355	0.35140	2.98468
2.5	0.19645	−0.61471	3.12906
2	0.24218	−0.77027	3.17053
1.5	0.31815	−1.02974	3.24669
1	0.46040	−1.54293	3.34128
0.75	0.61267	−2.04114	3.34157
0.5	0.93893	−3.02616	3.23297
0.25	1.93088	−6.07084	3.13208

**Table 4:** Ratio between tensile stress and splitting strength at the center of the specimen.

P (N)	Stress in the x-Direction at the Center *σ_x_* (MPa)	Splitting Strength *σ_IDT_* (MPa)	*σ_x_*/*σ_IDT_*
500	0.01253	0.01414	0.88574
1000	0.02506	0.02829	0.88574
1500	0.03759	0.04244	0.88574
2000	0.05012	0.05658	0.88574
4000	0.10024	0.11317	0.88575
5000	0.12530	0.14147	0.88575
10,000	0.25061	0.28294	0.88573
20,000	0.50123	0.56588	0.88576

**Table 5:** C35 concrete mix.

Concrete Mix Proportion (Cement–Water–Sand–Crushed Stone)	Concrete Material Quantities/m^3^ (kg/m^3^)					Maximum Coarse Aggregate Size (mm)
	Cement	Water	Sand	Crushed Stone	Water-Reducing Agent	
1:0.5:2:3.01	370	175	740	1115	1.48	20

**Table 6:** Results of splitting test.

Specimen	Splitting Tensile Strength *σ_IDT_* (MPa)	Average (MPa)	Unconfined Compressive Strength *σ_c_* (MPa)	Average (MPa)
C35	1.964	2.175	28.57	30.41
	2.247		30.47	
	2.314		32.19	

**Table 7:** Results of direct shear test.

Specimen	Normal Stress (Mpa)	Tangential Stress (Mpa)	Fitting Results
C35	1	6.16402	τ=1.451σ+4.812
	2	7.53186	
	3	9.34535	
	4	10.59337	

## Data Availability

The original contributions presented in this study are included in the article. Further inquiries can be directed to the corresponding author.
